# The diagnostic and prognostic value of D-dimer in different types of aortic dissection

**DOI:** 10.1186/s13019-022-01940-5

**Published:** 2022-08-20

**Authors:** Deli Wang, Jie Chen, Jianhua Sun, Hongmei Chen, Fang Li, Junfeng Wang

**Affiliations:** 1 Department of General Surgery, 904th Hospital of Joint Logistic Support Force of PLA (Suzhou), Suzhou, 215007 China; 2 Department of General Surgery, Suzhou BOE hospital, Suzhou, China; 3 Department of Nursing, 904th Hospital of Joint Logistic Support Force of PLA (Suzhou), Suzhou, 215007 China

**Keywords:** D-dimer, Diagnostic, Prognostic, Aortic dissection, Biomarker

## Abstract

**Objective:**

To evaluate the serum D-dimer level and its diagnostic and prognostic predictive value in patients with different types of aortic dissection.

**Methods:**

Eighty-four aortic dissection patients who were diagnosed clinically in our hospital from January 2017 to January 2021 were selected for the study. All patients were divided into Stanford type A (39 cases) and Stanford type B (45 cases) groups. The serum D-dimer level was detected at 1 h, 6 h, 12 h, 24 h, and 72 h after admission to the hospital, and its expression level with different types of aortic dissection was analyzed. The relationship between D-dimer and the prognosis of patients was also analyzed.

**Results:**

The serum D-dimer levels of patients in group A were significantly higher than those in group B at 6 h, 12 h, 24 h, and 72 h after admission, and the differences were statistically significant. In group A, 16 patients died, and 23 patients survived, while in group B, 18 patients died, and 27 patients survived. The serum D-dimer level of the dead and surviving patients in group A was significantly higher than that of group B, and the serum D-dimer level of dead patients in groups A and B was significantly higher than that of surviving patients. For diagnostic value, the AUC was 0.89, sensitivity was 76.92%, specificity was 90.00% in group A, and the AUC was 0.82, sensitivity was 71.11%, and specificity was 85.00% in group B. For the prognostic predicted value, the AUC was 0.74 in group A, while the AUC was 0.69 in group B.

**Conclusions:**

D-dimer has different serum levels in different types of aortic dissection patients, with higher levels in Stanford A. Serum D-dimer levels may be used as a better biomarker to diagnose the two types of aortic dissection and play an important role in patient prognostic prediction, especially Stanford type A.

## Introduction

Acute aortic dissection (AAD) is a life-threatening condition associated with a significant risk of mortality and morbidity. Mortality for AAD is 50% by 24 h, and 50% of patients die before reaching a specialist center [1, 2]. It is a serious cardiovascular disease caused by the tearing of the aortic middle layer and the invasion of blood into the aortic middle layer, resulting in a dissection hematoma. However, the specific pathogenesis of aortic dissection is not clear, and many studies have shown that genetic factors, hypertension, arteriosclerosis, aortic middle layer progressive degeneration, and aortic inflammation may be common pathogenic factors [3, 4]. According to the Stanford classification, aortic dissection can be divided into Stanford types A and B; type A mainly involves ascending aortic dissection, and type B involves the descending aorta and abdominal aorta far from the left subclavian artery [5]. The clinical classification of aortic dissection plays an important role in guiding diagnosis and treatment. However, regardless of the type of aortic dissection, all characteristics included rapid onset, rapid progression, changeable condition, and complex symptoms, which also increases the difficulty of clinical diagnosis. Aortic dissection is associated with high mortality if not diagnosed and treated immediately with surgical repair, the risk of disease progression and complications will be increased, and the life and health of patients will be seriously threatened [6, 7]. Therefore, it would be significant to the accuracy and rapidity of the clinical diagnosis of AAD, as it can reduce mortality and improve prognosis as early diagnosis and early treatment.

Chest CT, CTA, MRI, echocardiography, and other methods are usually used in the clinical diagnosis of AAD, while CT examination requires repeated movement of the patient, and MRI is time-consuming, has high technical requirements, and is limited by emergency room conditions compared to biomarkers for the diagnosis of AAD, especially in many primary hospitals [8]. An increasing number of studies have investigated potential AAD biomarkers for faster and more accurate clinical treatment, such as smooth muscle myosin, a calcium-binding protein, and D-dimers [9–11]. A recent meta-analysis reported that D-dimer is a useful tool for detecting suspected AAD and plays an important role in the auxiliary diagnosis of aortic dissection; And setting a cut-off value less than 500 ng/mL had better sensitivity to predict AD than a setting of D-dimer cur-off over 500 ng/mL [12]. Tokuda [13] also reported that serum biomarker detection can play an important role in acute and timely detection in the diagnosis of aortic dissection in the setting acute ischemic stroke or transient ischemic attack. Xie [14] reported that D-dimer ≥ 5.9 mg/L and type A AAD were independently associated with in-hospital mortality in AAD patients. Moreover, subgroup analysis proved that elevated D-dimer was related to poor prognosis in type A AAD. Hence, serum biomarker detection has the unique advantages of being non-invasive, rapid, simple, and economical in the diagnosis of aortic dissection, providing a new method for the diagnosis of active aortic dissection.

The present study, therefore, explored the diagnostic and prognostic value of D-dimer in different types of aortic dissection. To provide evidence for the clinical diagnosis, treatment, and prognostic prediction of aortic dissection.

## Methods

### Study design

This retrospective study followed the Equator guidelines. The reporting of this study conforms to the Strengthening the Reporting of Observational Studies in Epidemiology (STROBE) statement [15].

This was a retrospective study in Jiangsu between Jan 2017 and Jan 2021, and eighty-four aortic dissection patients were assessed. The study protocol was approved by the Anhui Medical University Affiliated Wuxi Clinical College Clinical Research Ethics Committees (2016-YXLL-092). The study protocol received Ethics Committee approval from all participating centers. Written informed consent was obtained from patients whose competence was established by their accurate orientation for time, place, and person, as well as an understanding of the recruiter’s description of the trial or otherwise from their next of kin or their legal representative. According to the examination results, they were divided into Stanford type A (group A with 39 cases) and Stanford type B (group B with 45 cases). All patients received serum D-dimer levels detected at 1 h, 6 h, 12 h, 24 h, and 72 h after admission to the hospital. The final follow-up was 90 days after admission.

### Study patients, inclusion criteria, and exclusion criteria

**Fig. 1:**
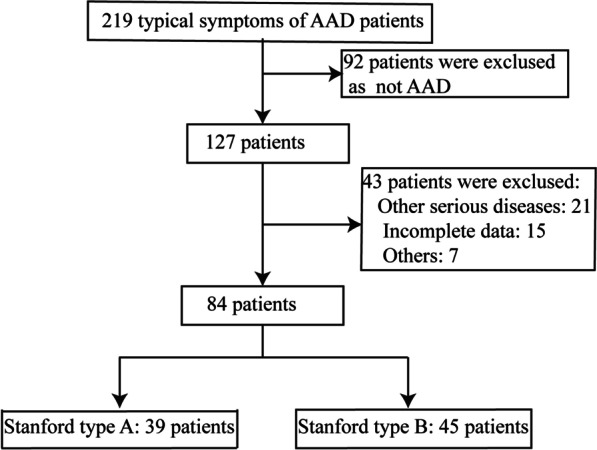
Trial profile. Note: other serious disease (21 cases) were exclused included Severe terminal disease with life expectancy < 6 months, primary pulmonary hypertension, venous thromboembolic disease, and severe valvular heart disease, cardiomyopathy, pericarditis. Others (7 cases) were exclused as family members give up treatment

A total of 84 eligible patients admitted to the hospital with acute chest disease and diagnosed with AAD from Jan 2017 to Jan 2021 were selected as the study subjects (Fig. 1). The inclusion criteria were as follows: (1) the clinical symptoms and signs of the patients were consistent with the typical symptoms of AAD; (2) AAD was confirmed by CT, MRI, or CTA after admission; (3) the patient had no history of AAD before admission, and the onset of symptoms was within 2 days; and (4) informed consent was signed for this study. Exclusion criteria were as follows: (1) previous history of AD, myocardial infarction, or recent surgery; (2) patients with severe valvular heart disease, cardiomyopathy, pericarditis, or primary pulmonary hypertension; (3) incomplete clinical data; and (4) researchers found other reasons.

### Diagnostic

The diagnostic criteria for AAD were confirmed by CT or aortic CTA examination, "double cavity sign" could be seen in the aorta during CT imaging, or damaged and stripped aortic intima could be seen in the aortic cavity. An aortic intima tear was observed during CTA examination, and the active veins were divided into true and false cavities. According to the above examination results, the diagnosis can be made by combining the clinical symptoms and auxiliary examination.

### Treatment and D-dimer detection

After admission, a venous blood test was collected for all patients, and relevant examinations were performed. After confirmation by CT or CTA, symptomatic treatment, such as analgesia and blood pressure control, was given. All cases present emergency surgery after preoperative examination immediately, AD surgery was performed for Stanford Type A patients, and endovascular stent intervention was performed for Stanford type B patients. The serum D-dimer level was detected at 1 h, 6 h, 12 h, 24 h, and 72 h after admission by ELISA.

### Statistical analysis

SPSS 19.0 statistical software (SPSS Institute, Hefei, Anhui Medical University) was used for the statistical analyses. All baseline and outcome data in the study database were collected and compared. All continuous variables are presented as the mean ± standard deviation. Independent two-sample t-tests and Spearman correlations were used to assess categorical data. Fisher’s exact t-test was used to compare categorical data between two groups, and the Mann‒Whitney U test was used to compare ordinal or continuous variables between groups. A receiver operating characteristic curve (ROC) was used to analyze the clinical diagnostic and prognostic prediction value of D-dimer for different types of AAD, and another twenty patients suspecting AD were randomly matched for receiver operating characteristic curve (Patients with negative). A value of *P* < 0.05 was considered statistically significant.

## Results

From Jan 2017 to Jan 2021, a total of 84 patients were divided into Stanford type A (39 cases) and Stanford type B (45 cases) groups. All type A AD patients received surgery and type B AD patients received endovascular treatments. The final visit of the last patient was performed on April 25, 2021. There were 25 males and 14 females in group A. The age ranged from 24 to 74 years, with an average age of 55.8 ± 11.4 years. There were 26 cases of hypertension, 12 cases of diabetes, and 24 cases of smoking. In group B, there were 27 males and 18 females. The age ranged from 26 to 76 years, with an average age of 56.3 ± 11.9 years. Hypertension was present in 29 cases, diabetes in 15 cases, and smoking in 27 cases. There was no statistically significant difference in baseline data between the two groups, including age, sex, history of hypertension, history of diabetes, history of smoking, and other basic information (Table 1, *P* > 0.05).

**Table 1:** Demographic and baseline characteristics of the study population in the two groups

Variable	A group	B group	P-value
Number of patients	39	45	
Age (mean ± SD)	55.8 ± 11.4	56.3 ± 11.9	0.845
Gender			0.699
Male	25 (64.1%)	27 (60.0%)	
Female	14 (35.9%)	18 (40.0%)	
Weight (kg)	58.4 ± 8.5	57.7 ± 8.1	0.704
History of hypertension			0.831
Yes	26 (66.7%)	29 (64.4%)	
No	13 (33.3%)	16 (35.6%)	
Coronary heart disease			0.719
Yes	3 (7.7%)	5 (11.1%)	
No	36 (92.3%)	40 (88.9%)	
Pain time	16.8 ± 6.8	15.2 ± 5.9	0.252
Nicotine use			0.886
Yes	24 (61.5%)	27 (60.0%)	
No	15 (38.5%)	18 (40.0%)	
Diabetes			0.802
Yes	12 (30.8%)	15 (33.3%)	
No	27 (69.2%)	30 (66.7%)	
Platelet counts (× 10^9^/L)	179.6 ± 33.4	183.5 ± 42.6	0.646
Fibrinogen (g/L)	4.34 ± 0.67	4.28 ± 0.55	0.653
APTT (s)	34.22 ± 3.15	33.79 ± 3.31	0.545
SBP (mmHg)	138.6 ± 23.8	137.1 ± 23.2	0.771
Heart rate (bpm)	84.6 ± 7.8	86.3 ± 8.3	0.334

*SBP* systolic blood pressure, *APTT* activated partial thromboplastin time

### Comparison of serum D-dimer in the two groups

All patients received serum D-dimer levels detected at 1 h, 6 h, 12 h, 24 h, and 72 h after admission. We found that the D-dimer levels significantly increased in the Stanford type A group at 6 h, 12 h, 24 h, and 72 h compared with the Stanford type B group (*p* < 0.05, Table 2).

**Table 2:** Comparison of serum D-dimer in two groups

Time	A Group (n = 39)	B Group (n = 45)	t value	*P* value
1 h	557.1 ± 263.1	461.3 ± 217.0	1.829	0.070
6 h	719.5 ± 184.2	622.8 ± 196.5	2.315	0.022
12 h	872.5 ± 211.4	762.8 ± 198.2	2.453	0.016
24 h	977.2 ± 261.5	827.2 ± 267.9	2.588	0.011
72 h	1426.8 ± 338.2	1254.8 ± 312.8	2.208	0.031

### Serum D-dimer levels and outcome

The final follow-up was 90 days after admission. Sixteen patients died in the Stanford type A group, and 18 patients died in the Stanford type B group. There were no surgical surprises. We chose 24-h D-dimer levels because the difference was most obvious. We found that the D-dimer levels (24 h) significantly increased in the death group compared with the survival group (*p* < 0.05, Table 3). Additionally, we also found that the D-dimer levels (24 h) significantly increased in the Stanford type A group compared with the Stanford type B group in the death group (*p* < 0.05, Table 3).

**Table 3:** Comparison of serum D-dimer levels in Stanford TYPE A and Stanford type B patients with death and survival (mg/L, x ± s)

	A Group (n = 39)	B Group (n = 45)	t value	*P* value
Death	1121.7 ± 283.9	910.6 ± 248.0	2.423	0.021
survival	886.8 ± 204.5	740.1 ± 265.3	1.816	0.078
t value	3.003	2.228		
*P* value	0.005	0.03		

### The diagnostic value of D-dimer in different types of aortic dissection

**Fig. 2:**
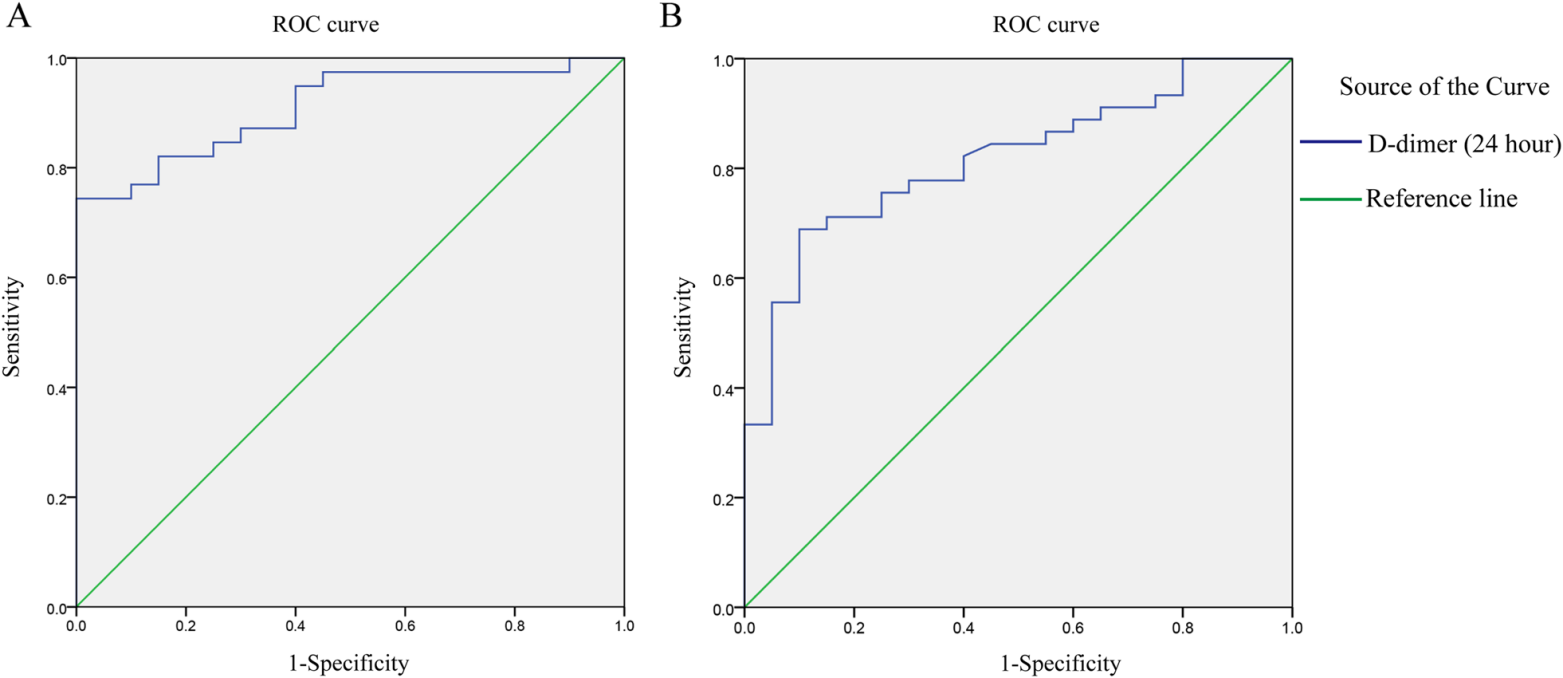
The diagnostic value of D-dimer in different types of aortic dissection. **A** Stanford Type A aortic dissection, ROC curve AUC = 0.89 (*p* < 0.001), sensitivity = 76.92%, specificity = 90.00%, the cutoff value of D-dimer was 422.5. **B** Stanford Type B aortic dissection, ROC curve AUC = 0.82 (*p* < 0.001), sensitivity = 71.11%, specificity = 85.00%, the cutoff value of D-dimer was 442

To explore the diagnostic value of D-dimer (1 h) in different types of aortic dissection, we used the ROC curve analytical method, which showed that the area under the ROC curve AUC was 0.89 (*p* < 0.001), the sensitivity was 76.92%, and the specificity was 90.00%, the cutoff value of D-dimer was 422.5. The area under the ROC curve (AUC) was 0.82 (*p* < 0.001), the sensitivity was 71.11%, and the specificity was 85.00%, the cutoff value of D-dimer was 442 (Fig. 2).

### The prognostic prediction value of D-dimer in different types of aortic dissection

**Fig. 3:**
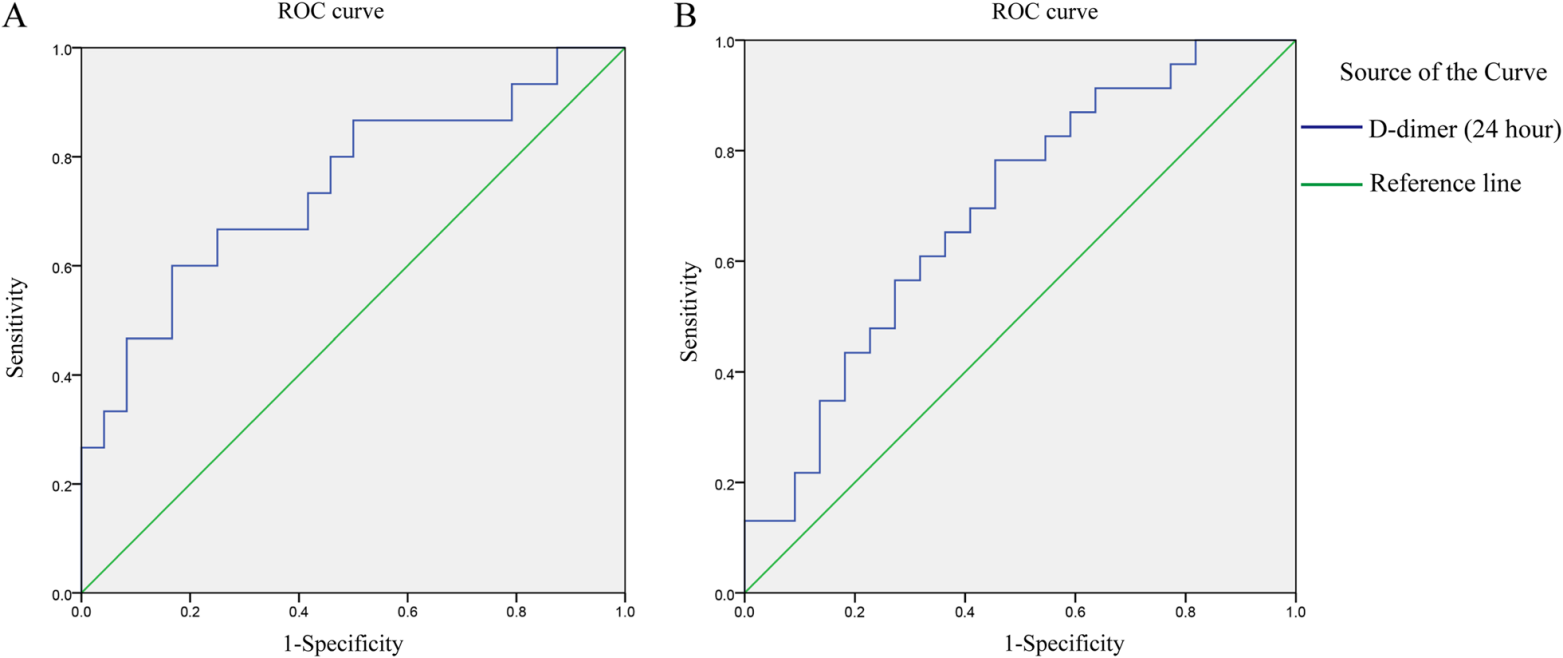
The prognostic prediction value of D-dimer in different types of aortic dissection. **A** Stanford Type A aortic dissection, ROC curve AUC = 0.74 (*p* = 0.010), the cutoff value of D-dimer was 919.5. **B** Stanford Type B aortic dissection, ROC curve AUC = 0.69 (*p* = 0.028), the cutoff value of D-dimer was 835.5

We also used the ROC curve analytical method to explore the prognostic prediction value of D-dimer (24 h) in different types of aortic dissection, which showed that the area under the ROC curve AUC was 0.74 (*p* = 0.010), the cutoff value of D-dimer was 919.5 in the Stanford Type A aortic dissection. The area under the ROC curve (AUC) was 0.69 (*p* = 0.028), the cutoff value of D-dimer was 835.5 in the Stanford Type B aortic dissection (Fig. 3).

## Discussion

Aortic dissection has a rapid onset and progression, and its clinical manifestations are easily similar to those of cardiovascular diseases such as acute myocardial infarction, leading to a high misdiagnosis rate and clinical diagnosis difficulties. A ruptured aortic dissection can lead to shock or even death if misdiagnosis or delayed diagnosis occurs [16]. Previous studies have shown that the mortality rate of aortic dissection within 48 h is approximately 30% and as high as 50% at 14 d [17]. Therefore, it would greatly save the lives of patients with aortic dissection after enhancing the early, rapid, and accurate diagnosis of aortic dissection. Imaging has always been regarded as the only means of clinical diagnosis of aortic dissection, but imaging examination is time-consuming, and some patients have contraindications for CT and MRI examination, limiting the application of imaging in the diagnosis of aortic dissection. An increasing number of doctors and studies have reported that serum biochemical markers can be used for the clinical diagnosis of aortic dissection [18, 19]. According to the findings of the current investigation, D-dimer was an important biomarker in the diagnosis and prognostic prediction of different types of aortic dissection. The diagnostic and predictive values were better in Stanford type A.

D-dimer is the degradation product of human crosslinked fibrin, which plays an important role in the body's anticoagulant system, and it is also one of the key substances to maintain human blood vessel wall permeability and normal blood flow. D-dimer is involved in the process of coagulation and fibrinolysis, so it is often regarded as a serum marker reflecting coagulation and fibrinolysis in patients. The D-dimer can reflect the fibrinolysis process of coagulation, and its level is related to the size of thrombus formation and the contact area between the thrombus and blood [20]. D-dimer levels were also of great prognostic significance and were associated with outcomes in patients with vascular diseases [21, 22]. Kida [23] also reported that D-dimer levels were a biomarker for predicting ischemic stroke, below the reference value in patients with nonvalvular atrial fibrillation and acute heart failure. Additionally, Hisamitsu [24] demonstrated that a high D-dimer concentration may predict a worse prognosis in patients undergoing acute endovascular cerebral thrombectomy. A retrospective observational cohort study also confirmed that D-dimer plays an important role in acute kidney injury in living donor liver transplantation [25]. In pulmonary embolism disease, thrombus burden was related to elevated D-dimer levels, and D-dimer values > 1.18 mg/l were predictive of right ventricular dysfunction in normotensive patients. D-dimer levels were influenced by deep venous thrombosis but were not influenced by cancer, pneumonia, age, or renal impairment [26]. The inflammatory response was activated after the onset of cardiovascular disease and then induced the activation of endogenous coagulation and fibrinolysis continuously, which resulted in increased serum D-dimer. Hence, elevated D-dimer may play an important role in the formation and development of various cardiovascular diseases.

The present study analyzed the serum D-dimer levels of patients with different types of aortic dissection and showed that the serum D-dimer level of Stanford type A patient was significantly higher than that of Stanford type B patients at 1, 6, 12, 24, and 72 h after admission. Serum D-dimer levels were different in patients with different types of aortic dissection, which provides a new reference for the clinical classification of aortic dissection. Additionally, the results showed that the serum D-dimer level of Stanford type A was significantly higher than that of Stanford type B in the death and survival patients, and the serum D-dimer level of Stanford type A and Stanford type B death patients was significantly higher than that of surviving patients. The reason may be that Stanford type A aortic dissection involves the ascending aorta, leading to an easier pseudolumen opening. Then, the high-speed flowing blood in the ascending aorta continues to tear the intima of the vessel, resulting in continuous coagulation and fibrinolysis reactions on the contact surface of the middle aorta. Therefore, the level of D-dimer in Stanford type A patient was higher than that in Stanford type B patients. Keskin [27] also reported that D-dimer was an independent indicator or biomarker to predict in-hospital mortality. The ROC analysis in the present study also showed that D-dimer plays an important role in diagnostic and prognostic prediction in patients with different types of aortic dissection.

This study had several limitations that need to be improved upon. Additional clinical factors should be examined in baseline data. Additionally, this is a small sample size in a single center, and a multicenter, large sample study is needed to evaluate the effectiveness of this treatment.

## Conclusion

The findings of the present research suggest that the levels of D-dimer were different in Stanford A and Stanford B patients with aortic dissection at different periods. Overall, the level of Stanford A serum dimer was higher than that of Stanford B serum dimer. The detection of serum D-dimer levels in patients can help to determine the classification of aortic dissection, which has important application value for clinical diagnosis and treatment. However, due to the limited sample size of this study, the conclusion of this study had some limitations. Therefore, it is still necessary to expand the sample size in the future to explore the diagnostic efficacy of serum D-dimer in aortic dissection.

## Data Availability

The corresponding author may provide the datasets used in the present research upon request.
